# Ultrasound therapy for a week promotes regeneration and reduces pro-inflammatory macrophages in a rat sciatic nerve autograft model

**DOI:** 10.1038/s41598-023-38630-8

**Published:** 2023-07-17

**Authors:** Hideki Kawai, Akira Ito, Asuka Kawaguchi, Momoko Nagai-Tanima, Ryo Nakahara, Shixuan Xu, Hiroshi Kuroki

**Affiliations:** 1 Department of Motor Function Analysis, Human Health Sciences, Graduate School of Medicine, Kyoto University, 53 Kawahara-cho, Shogoin, Sakyo-ku, Kyoto, 606-8507 Japan; 2 Japan Society for the Promotion of Science, Tokyo, Japan

**Keywords:** Regeneration and repair in the nervous system, Orthopaedics, Peripheral nervous system, Rehabilitation

## Abstract

Peripheral nerve injury causes long-term motor dysfunction. Ultrasound (US) therapy is expected to accelerate peripheral nerve regeneration. However, its optimal usage and effects on macrophage phenotypes during peripheral nerve regeneration remain unknown. In this study, we investigated the optimal duration of US therapy and its effects on macrophage phenotype. Twenty-seven rats with autologous sciatic nerve grafting were divided into three groups: two received US therapy (1 MHz frequency, intensity of 140 mW/cm^2^, 20% duty cycle, 5 min/day) for one (US1) or 4 weeks (US4), and one group received sham stimulation. Immunohistochemistry was performed 3 and 7 days after injury in another set of 12 rats. Eight weeks after the injury, the compound muscle action potential amplitude of the gastrocnemius in the US1 and US4 groups was significantly higher than that in the sham group. The toe-spreading test showed functional recovery, whereas the gait pattern during treadmill walking did not recover. There were no significant differences in motor function, histomorphometry, or muscle weight between groups. Immunohistochemistry showed that US therapy decreased the number of pro-inflammatory macrophages seven days after injury. Therefore, US therapy for both one or 4 weeks can similarly promote reinnervation and reduce proinflammatory macrophages in autograft model rats.

## Introduction

Peripheral nerve injuries lead to motor dysfunction, particularly in cases with long nerve defects^1^. Injured peripheral nerves regenerate spontaneously; however, degeneration of the neuromuscular junction or muscle atrophy caused by long-term denervation or misdirection of regenerating axons inhibits motor function recovery, even if the axons reach the endplate^2,3^ Therefore, approaches for promoting regeneration need to be developed.

Macrophages play important roles in axonal regeneration. After injury, macrophages are recruited to the injury site to phagocytose degenerated axons and myelin debris^4^. Furthermore, macrophages can secrete several proteins, such as axon guidance molecules, neurotrophic factors, and proteases, to support axon regeneration^5–7^. Depending on their activity and protein expression, infiltrating macrophages are divided into two major phenotypes: pro-inflammatory (M1 macrophages) and anti-inflammatory (M2 macrophages)^8^. The number of M1 macrophages increases soon after injury, and that of M2 macrophages increases afterward^9^. Peripheral nerve regeneration can be inhibited and promoted by modulating macrophage polarization to M1 and M2, respectively^10^. These results imply that macrophages can be a therapeutic target and that the timing of therapy is crucial for promoting peripheral nerve regeneration.

Ultrasound (US) is a potential therapeutic modality for treating peripheral nerve injuries. US therapy is applied to many tissues such as bones, tendons, and cartilage^11^. Mechanical stimulation transmitted by pulsed US causes intracellular signal transduction via cell adhesion molecules, such as integrins and mechanosensitive ion channels, and can promote tissue healing^12,13^. US promotes peripheral nerve regeneration in crush injuries, autologous nerve grafts, and cell-seeded graft model rats^14^. The optimal parameters for US therapy, especially intensity, have been explored in several studies for effective treatment of peripheral nerve injury^15–17^. We previously found that US therapy initiated the day after injury rather than after a delay promotes peripheral nerve regeneration in a rat sciatic nerve crush injury model^18^, indicating that the timing of US therapy is also essential. The duration of US therapy must be optimized for further peripheral nerve regeneration. Moreover, when macrophages are affected by US stimulation, phagocytosis is accelerated, and their phenotypes are modulated^19,20^. Although the effects of US on macrophages during peripheral nerve regeneration have not yet been revealed, these results indicate that US therapy in the early phase, especially in the first week when macrophages translate from M1 to M2^9^ after peripheral nerve injury, promotes regeneration by affecting macrophage activation.

Additionally, our previous study did not show significant differences in motor function recovery after spontaneous regeneration^18^. Therefore, more severe injury models are required to evaluate the effects of US on motor function recovery. In the current study, we used an autograft model as a more severe model than a crush injury, which led to insufficient motor function recovery^21^.

This study aimed to investigate the optimal duration of US therapy and its effects on macrophages in an autografted rat sciatic nerve model.

## Results

### Motor functional recovery and gait pattern alteration after injury

**Figure 1:**
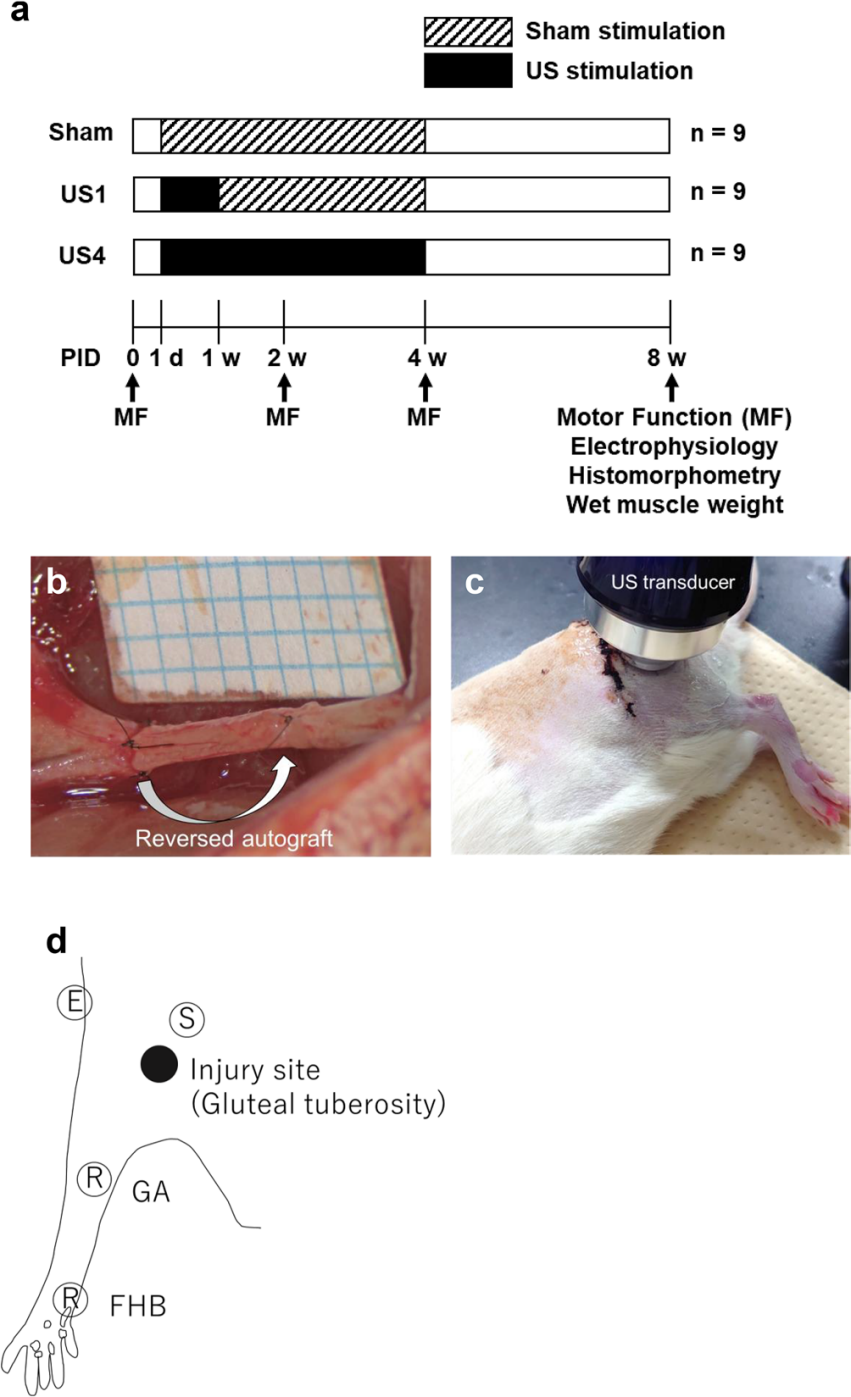
Study design and experimental settings. (**a**) Scheme of ultrasound (US) therapy and evaluation of its optimal duration for US therapy. (**b**) A 5-mm-long sciatic nerve was excised and autografted in a reversed orientation. (**c**) US transducer placed on skin above the injury site. (**d**) Stimulating and recording site of the electrophysiological study. The rats were placed in the prone position, and each electrode was inserted percutaneously. S, stimulating electrode; R, recording electrodes for the gastrocnemius (GA) and flexor hallucis brevis (FHB); E, earth electrode; filled circle, injury site**.** PID, post-injury day.

**Figure 2:**
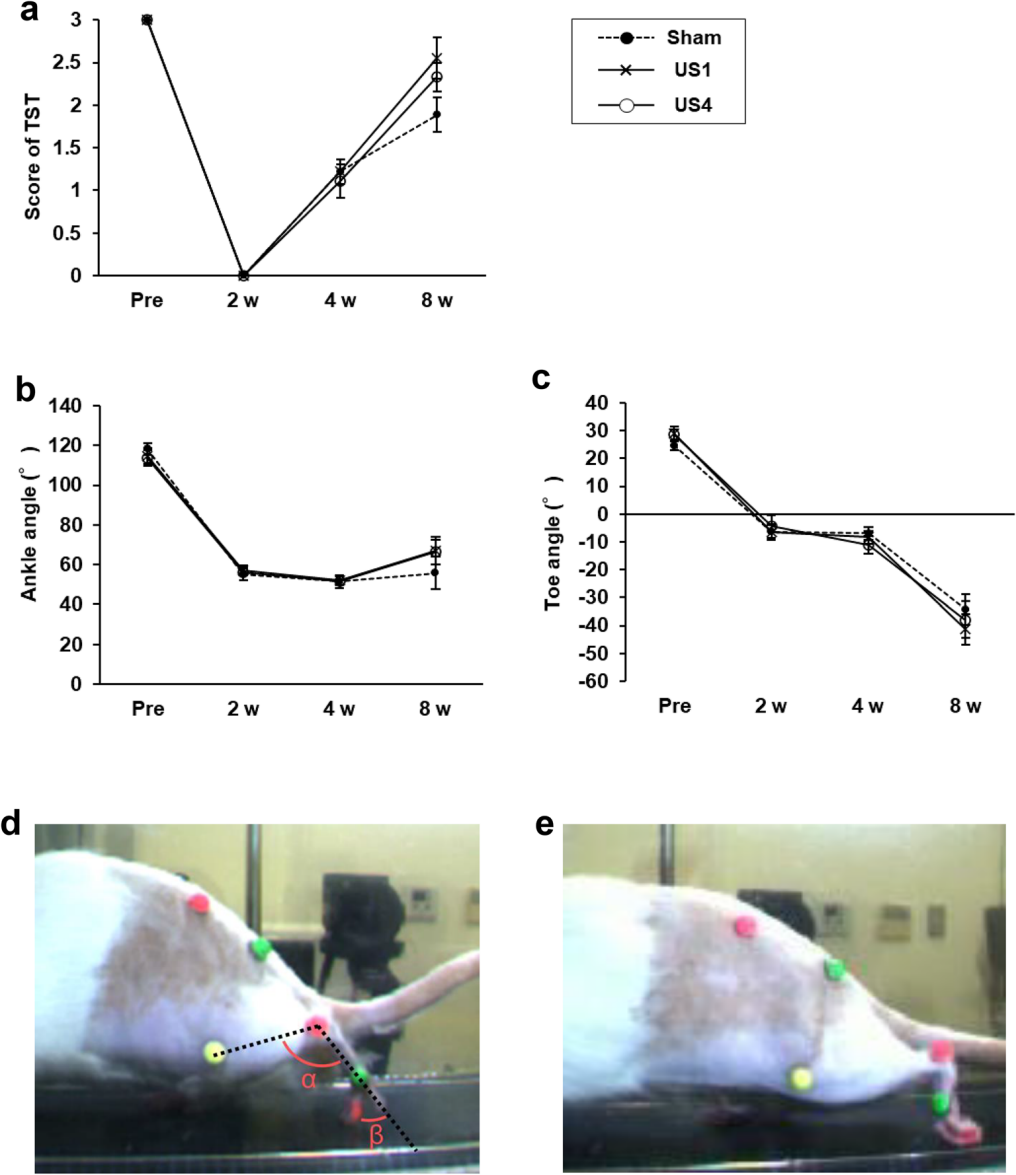
Recovery of motor function with no recovery in gait patterns. (**a**) Toe-spreading test (TST), and (**b**) ankle and (**c**) toe angle in the toe-off phase during treadmill walking performed as motor function analyses preoperatively and at 2, 4, and 8 weeks after injury. The result of TST showed the recovery of motor function, while the gait patterns did not recover. (**d**) The ankle (α) and toe angle (β) in the toe-off phase before the injury and (**e**) at 8 weeks after injury. Data are expressed as the mean ± standard error (n = 9 for each group).

After the sciatic nerve was autografted, the rats received US therapy for one (US1 group) or four weeks (US4 group), or received sham stimulation (Fig. 1). Motor function recovery was assessed before injury and at 2, 4, and 8 weeks after injury. Self-mutilation of the foot or any adverse events were not observed. The results of the toe-spread test showed motor functional recovery over time (Fig. 2a); however, there were no significant differences between the groups (sham group, 1.89 ± 0.20; US1, 2.56 ± 0.15; US4, 2.33 ± 0.17). Motor function during treadmill walking was analyzed using a three-dimensional motion capture system (Fig. 2b–e). Each marker attached to the landmarks was traced in a video recorded from two different directions, and the ankle and toe angles were reconstructed in three dimensions. The ankle and toe angles in the toe-off phase decreased after the injury. The mean ankle angle of the US1 and US4 groups at 8 weeks showed a slight increase compared with that at 4 weeks; however, there were no significant differences in the mean ankle angles between the groups at 8 weeks (Fig. 2b; sham group, 55.74° ± 8.11°; US1 group, 66.91° ± 6.96°; US4 group, 66.39° ± 6.34°). In contrast, the toe angles continued to decrease from 4 to 8 weeks (Fig. 2c). There were no significant differences in the mean toe angles between the groups at 8 weeks (sham group, − 34.22° ± 5.57°; US1 group, − 41.33° ± 5.48°; US4 group, − 37.93° ± 6.50°). The preoperative rats showed toe extension during the stance phase and terminal swing (Fig. 2d, Supplementary Fig. S1, Supplementary Video S1), whereas all rats showed excessive toe flexion in all step cycle phases at 8 weeks after injury (Fig. 2e, Supplementary Video S2).

### Enhancement of reinnervation by US therapy

**Figure 3:**
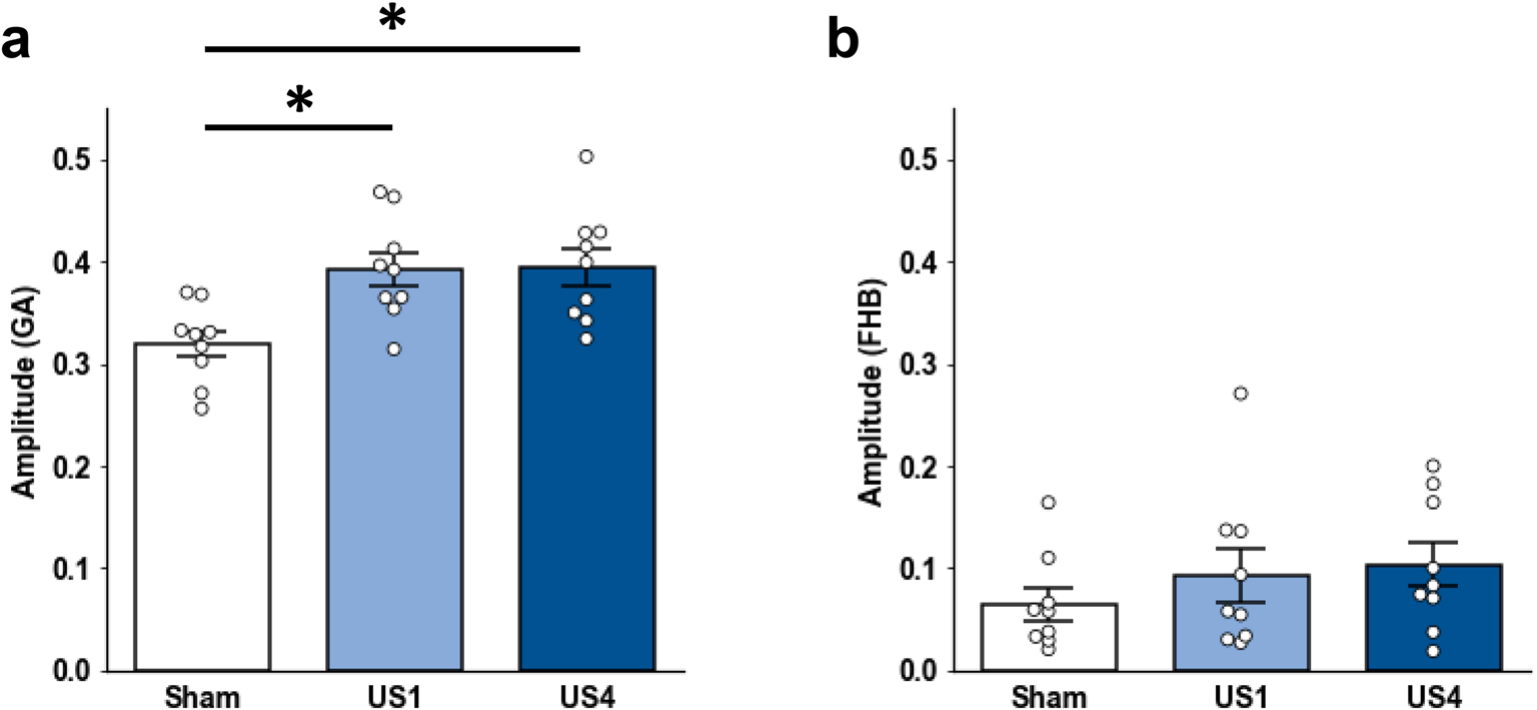
Reinnervation promoted by ultrasound therapy. (**a**) The amplitudes of compound muscle action potentials of the gastrocnemius (GA) and (**b**) flexor hallucis brevis (FHB) expressed as the ratio of the injured side to the non-injured side. Data are expressed as the mean ± standard error. Each plot shows individual values (n = 9 for each group). *P < 0.05.

The compound muscle action potentials (CMAPs) of the gastrocnemius (GA) and flexor hallucis brevis (FHB) muscle, an intrinsic muscle of the feet, were assessed to confirm reinnervation (Fig. 1d). The CMAP amplitudes of the GA in the US1 (0.39 ± 0.05) and US4 (0.39 ± 0.06) groups were significantly higher than those of the sham groups (Fig. 3a; 0.32 ± 0.04; *P* = 0.0358 and 0.0282, respectively). The GA amplitude correlated with the ankle angle (Spearman’s *ρ* = 0.4109, *P* = 0.0333). Moreover, the amplitudes of the FHB were also detected in all groups (sham group, 0.06 ± 0.16; US1 group, 0.09 ± 0.03; US4 group, 0.10 ± 0.02); however, there were no significant differences (Fig. 3b). The amplitude of the FHB was positively and negatively correlated with the ankle (Spearman’s *ρ* = 0.5024, *P* = 0.0076) and toe (Spearman's *ρ* = − 0.4792, *P* = 0.00114) angles at 8 weeks, respectively.

### No significant differences in histomorphometry and muscle weight analysis

The diameters of the myelinated nerve fibers and axon, the thickness of the myelin sheath, and the density of the myelinated nerve fibers were measured from semi-thin and ultrathin sections at the mid-portion of the graft to assess the effects of US on histological regeneration (Supplementary Fig. S2). However, there were no significant differences between the groups. Muscle mass recovery was also assessed. The results of wet muscle weight analysis showed atrophy of the injured side in all assessed muscles 8 weeks after injury (Supplementary Fig. S3). There were no significant differences between the groups.

### Modulation of macrophage phenotypes by US therapy

**Figure 4:**
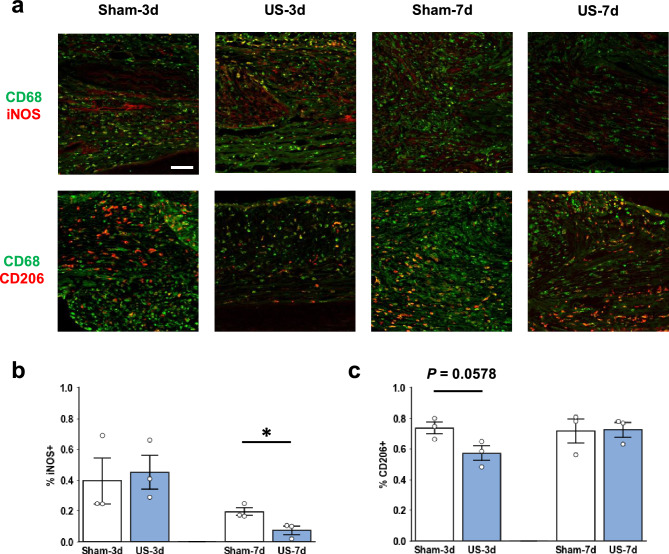
The phenotype of macrophages modulated by ultrasound therapy. (**a**) Representative images of CD68 positive macrophages (green) co-expressing inducible nitric oxide synthase (iNOS) or CD206 (red). (**b**) The ratio of macrophages expressing iNOS (% iNOS +) or (**c**) CD206 (% CD206+). Data are expressed as the mean ± standard error. Each plot shows individual values (n = 3 for each group). Scale bar = 100 μm. *P < 0.05.

Another 12 rats were used for immunohistochemical staining analysis to investigate the effects of US on the phenotypes of macrophages at 3 and 7 days after injury, when translation from M1 to M2 occurred^9^. The CD68 positive macrophages co-expressing inducible nitric oxide synthase (iNOS) and CD206 were identified as M1 and M2 macrophages, respectively (Fig. 4a). At 7 days after injury, the ratio of iNOS-positive macrophages (% iNOS +) in the US group (0.07 ± 0.03) was significantly decreased compared with that in the sham group (Fig. 4b; 0.19 ± 0.05, *P* = 0.0035). At 3 days after injury, fewer CD206 positive macrophages (% CD206 +) were observed in the US group (0.57 ± 0.05) compared with the sham group (0.74 ± 0.04); however, the difference was not significant (Fig. 4c  P = 0.0578).

## Discussion

The effects of US therapy on peripheral nerve injury are promising. However, its optimal usage and regeneration-promoting mechanisms remain unclear. In the present study, we investigated the effects of US on macrophages and determined the optimal duration of US therapy. We also performed three-dimensional motion analysis to evaluate motor function recovery in a sciatic nerve autograft rat model.

First, we investigated the optimal duration of US therapy and revealed that one-week or four-week therapy increased the CMAP amplitude of the GA muscle. The amplitude of CMAPs correlates with the fully innervated neuromuscular junction^22^, indicating that an increased CMAP amplitude mirrors muscle reinnervation. This also explains why the amplitudes of the FHB muscles, placed distal to the GA muscles, were approximately 0.1 even after US therapy, as few axons reached the neuromuscular junction. Combined with our previous study, which revealed that US therapy initiated the day rather than a week after injury promoted regeneration in rats with sciatic nerve crush injury^18^, our current results indicate that US therapy for a week after injury is essential for peripheral nerve regeneration. Second, we investigated the effects of US on macrophage phenotypes after injury. The modulation of macrophage phenotypes is a strategy for promoting peripheral nerve regeneration. A previous study showed that IL-4 or IFNγ injection into a nerve conduit modulates macrophage phenotypes and promotes or inhibits nerve regeneration^18^. Also, US stimulation can modulate phenotypes of macrophages in vivo and in vitro^20,23^. Our immunohistochemistry results showed that the percentage of iNOS + macrophages was lower in the US group than in the sham group 7 days after injury, indicating that M1 macrophages were decreased by US therapy during the early phase of inflammation. This result is consistent with those of previous studies showing that US therapy decreases iNOS expression after spinal cord or muscle injury^20,23^, similar to our previous study showing that US therapy suppresses inflammatory genes 7 days after sciatic nerve crush injury^17^. M1 macrophages are required for the clearance of myelin debris^24^. Myelin debris contains molecules that inhibit axon regrowth^25^; therefore, a delay in Wallerian degeneration inhibits peripheral nerve regeneration. On the other hand, M1 macrophages also inhibit peripheral nerve regeneration. A previous study that polarized macrophages forward to the M1 phenotype by employing cytokines revealed that prolonged high expression of M1 macrophages inhibited axonal regrowth^10^. Macrophages polarization mainly occurs within one week after injury^9^. Our electrophysiological results showed that US therapy for a week after injury promoted reinnervation, indicating that the effect of US therapy on macrophage phenotypes is a mechanism promoting peripheral nerve regeneration.

Incomplete motor function recovery after severe peripheral nerve injury highlights the importance of functional assessments. The sciatic functional index (SFI), an analysis of footprints on walking tracks, is widely and traditionally used as a quantitative assessment of functional recovery after sciatic nerve injury^26^; however, toe contracture or self-mutilation of the foot makes it impossible to obtain footprints^27,28^. Although we did not observe such a deterioration, some rats walked without the soles of the injured foot in contact with the ground (Supplementary Video S2). This gait pattern prevented us from obtaining footprints to analyze SFI. Instead, we performed three-dimensional motion analysis to assess motor function recovery. We previously reported that the toe angles in the toe-off phase during treadmill walking recovered as the nerves regenerated in a sciatic nerve crush injury model rats^29^. In contrast, our current study on a sciatic nerve autograft rat model did not show toe angle recovery, even in rats that received US therapy, although the gait pattern changed over time. Compared to our previous study on rats with crush injury rats^18^, whose toe angle recovered spontaneously, there was little difference in the CMAP amplitude of the GA and muscle weight. In addition, the toe angles did not recover even at 18 weeks after injury when CMAP amplitudes and muscle weights were increased compared to those at 8 weeks (Supplementary Fig. S4). The toe angle was also negatively correlated with FHB amplitude. These results indicate that the axons regenerated sufficiently to reestablish motor function and that the alteration of the gait pattern is a result of reinnervation. The results of the toe-spread test showed spontaneous functional recovery (Fig. 2a). Thus, motor function analysis needs to focus not only on whether rats can move but also on how they move.

Our study has several limitations. In this study, we observed significant differences only in the CMAP amplitudes of the GA. A previous study revealed that US therapy intensities of 250 or 500 mW/cm^2^ (spatial average temporal average, the same applies hereafter) were effective for histological regeneration in a sciatic nerve autograft model^16^. We used a US intensity of 140 mW/cm^2^ as the optimal intensity in a sciatic nerve crush injury rat model^17^. The optimal intensity might differ depending on the type of injury; however, a US therapy intensity of 140 mW/cm^2^ is sufficient to enhance reinnervation. Further studies are required to optimize the ultrasonic parameters of the autograft model. In addition, the current study did not demonstrate any effect of US on motor functional recovery. Even if reinnervation is enhanced, the misdirection of regenerating axons may inhibit voluntary behavior^30^. Approaches for enhancing axon regeneration to reinnervate appropriate targets, modulate neuroplasticity, and accelerate axon regeneration need to be considered. We observed no differences in the histomorphometry results. There were few differences between 8 and 18 weeks after injury, indicating that the nerves at the injury site had already regenerated in the sham group. To confirm the effect of US on accelerating axonal regeneration, histological regeneration of the distal portion of the graft and the injury site needs to be evaluated. In addition, we did not examine the effects of M1 macrophages on peripheral nerve regeneration. However, the mechanisms underlying the influence of M1 macrophages on peripheral nerve regeneration require further clarification. Finally, the sample size for immunohistochemical analysis was small. Although there is little doubt that US therapy can modulate macrophage phenotypes, some of the effects of US may have been overlooked. While the percentage of iNOS + macrophages decreased from 3 to 7 days, the percentage of CD206 + macrophages did not change in the sham group in the current study. A previous study that used a polycaprolactone conduit to graft 15-mm-long sciatic nerves showed that the percentage of CD206 + macrophages increased from 3 to 14 days after grafting^31^. We used a 5-mm-long autograft model rat. Differences in the length and material of the graft may affect the timing of macrophage polarization. Additionally, some macrophages may express both M1 and M2 markers^24^; therefore, a clear separation of M1 and M2 macrophages is difficult. Further studies are needed to determine the temporal changes in macrophage phenotypes using various grafting models.

In conclusion, US therapy for both one and four weeks after injury similarly promoted reinnervation and reduced pro-inflammatory macrophages in the sciatic nerve autografted rat model.

## Methods

### Animals

A total of 39 eleven-week-old male Lewis rats weighing 230–280 g (Shimizu Laboratory Supplies Co., Ltd., Kyoto, Japan) were purchased and dual-housed in standardized cages with water and food ad libitum under a 12 h light/dark cycle. Twenty-seven rats were used to investigate the optimal duration of US therapy and were randomly assigned to three treatment groups: sham (sham, n = 9), one-week (US1 group, n = 9), and four-week (US4, n = 9; Fig. 1a). Another 12 rats were divided into sham (n = 6) or US (n = 6) groups and used for immunohistochemistry 3 or 7 days after injury. The sample size was determined based on previous studies^18,32^. All procedures were approved by the Institutional Animal Care and Use Committee of Kyoto University (MedKyo 21577) and performed in accordance with the Regulations on Animal Experimentation at Kyoto University and the ARRIVE guidelines.

### Surgery

All rats underwent sciatic nerve autograft surgery one week after being raised. The rats were anesthetized with an intraperitoneal injection of a mixed anesthetic (0.15 mg/kg medetomidine, 2 mg/kg midazolam, 2.5 mg/kg butorphanol). The left sciatic nerve was exposed via a longitudinal lateral incision along the thigh. A five-mm-long sciatic nerve was excised at the mid-thigh, 85 to 90 mm from the tip of the third toe, and grafted in a reversed orientation with 9-0 nylon sutures (T06A09N20-25, Bear Medic Corporation; Fig. 1b). The incision was closed with 4-0 nylon sutures (S15G04N-45, Bear Medic Corporation), and 0.375 mg/kg atipamezole was administered intraperitoneally to reverse the anesthesia.

### US treatment

US treatment was performed using an ultrasonic treatment apparatus (UST-770, ITO Co., Ltd., Tokyo, Japan) according to our previous work^17,18^. This apparatus can be used as low-intensity pulsed ultrasounds. The US irradiation was applied under the same conditions, except when the US was applied. The rats were anesthetized with 2% isoflurane, and a US transducer (effective radiation area: 0.9 mm^2^, beam non-uniformity: 2.9) was placed on the skin above the injury site through a coupling gel (Fig. 1c). We applied ultrasound at the proximal end of the graft, and the site in the injured axons initiated elongation, using the gluteal tuberosity as a landmark. The US parameters were as follows: acoustic frequency, 1 MHz; intensity, 140 mW/cm^2^ (spatial average temporal average); repetition frequency, 1 kHz; duty cycle, 20%; and irradiation time, 5 min/day. All rats received daily US or sham US treatment (US treatment without US emission) for the first week after surgery, and then five times per week for three weeks.

### Motor functional analysis

#### Toe-spread test

The toe spread test was conducted as described in a previous study^33^. Toe movement during tail suspension was graded as follows: Grade 0, no toe movement; Grade 1, any sign of toe movement; Grade 2, toe abduction; and Grade 3, toe abduction with extension.

#### Gait analysis

Motor functional analysis was conducted using a three-dimensional motion capture apparatus (Kinema Tracer System, Kissei Comtec Co., Ltd., Nagano, Japan) preoperatively and at 2, 4, and 8 weeks after surgery according to our previous study^29^. The rats were anesthetized with 2% isoflurane, and hemispheric markers were attached to the following bilateral landmarks: the anterior superior iliac spine, greater trochanter, knee, lateral malleolus, and fifth metatarsophalangeal. The fourth toes were colored with pink ink. The rats walked on a treadmill at a speed of 12 m/min, consisting of more than five consecutive steps, and were recorded using four CCD cameras at 120 frames per second. Each marker was traced automatically, and the mean angles of the ankle and toe in the toe-off phase in ten-step cycles were analyzed (Fig. 2d).

### Electrophysiological study

Following motor functional analysis at 8 weeks, the rats were anesthetized with mixed anesthetics, and electrophysiological analysis was performed using an electromyogram measuring system (Neuropack S1 MEB-9404; Nihon Kohden, Tokyo, Japan), according to previous studies^18,34^. Sciatic nerves were stimulated proximal to the injury site, and CMAPs were detected in the GA, innervated by the tibial nerve, and the FHB muscles, innervated by the medial plantar nerve (Fig. 1d). The amplitude of CMAPs was expressed as the ratio of the injured side to the non-injured side.

### Histomorphometry analysis

After electrophysiological analysis, the rats were euthanized under deep isoflurane anesthesia, and the left sciatic nerve was dissected. Semithin and ultrathin transverse sections were prepared in the middle of the graft according to a previous study^29^. Images of semithin sections stained with toluidine blue were obtained using a light microscope (ECLIPSE 80i; Nikon, Tokyo, Japan). The number of myelinated nerve fibers was counted using the ImageJ software (National Institutes of Health, Bethesda, MD, USA), and the density was calculated. Ten areas of each ultrathin section stained with uranyl acetate and lead citrate were randomly obtained at 2000-fold magnification using a transmission electron microscope (TEM; H-7000, Hitachi High-Technologies, Tokyo, Japan). The shortest diameter of the myelinated nerve fibers (α) and axons (β) was measured using the ImageJ software, and the myelin sheath thickness of each fiber was calculated using the formula (α − β)/2.

### Wet muscle weight measurement

After sciatic nerve dissection, the tibialis anterior, extensor digitorum longus, GA, soleus (Sol), and FHB muscles were dissected and weighed. The results were expressed as the ratio of the injured side to the non-injured side. The FHB muscles of 9 rats were not assessed because of sample loss.

### Immunohistochemistry

The left sciatic nerve was fixed with 4% paraformaldehyde, cryoprotected with 30% sucrose, and 10 μm-thick longitudinal sections were prepared. The sections were washed with phosphate-buffer saline (PBS) and incubated with blocking buffer containing 5% goat serum for an hour. Following the blocking procedure, primary antibodies diluted in blocking buffer were applied overnight at 4 °C. The following primary antibodies were used: mouse anti-CD68 (1:500; MCA341GA; Bio-Rad Laboratories Inc. Hercules, CA, USA), rabbit anti-iNOS (1:200; ab15323; Abcam, Cambridge, UK), and rabbit anti-CD206 (1:500; #24595; Cell Signaling Technology, Danvers, MA, USA). After washing with PBS, secondary antibodies, Alexa Fluor 488-conjugated goat anti-mouse IgG (1:500, #A11001; Thermo Fisher Scientific, Waltham, MA, USA) and Alexa Fluor 594-conjugated goat anti-rabbit IgG (1:500, #A11012; Thermo Fisher Scientific), were applied for 30 min at room temperature. The slides were washed with PBS, and an autofluorescence quenching kit with DAPI (#SP-8500; Vector Laboratories, Newark, CA, USA) was used. Images were obtained using a confocal laser scanning microscope (FV10i; Olympus, Tokyo, Japan). Two images around the graft were randomly captured using a 20 × objective lens, and the number of macrophages was counted. CD68-positive cells with DAPI were counted as macrophages, and macrophages expressing iNOS and CD206 were counted as M1 and M2 macrophages, respectively^32^. The images were blinded so that the analyzer was unaware of the group and time point, and the number of cells was counted manually.

### Statistical analysis

Data are expressed as mean ± standard error. All statistical analyses were performed using JMP Pro 15.2 (SAS Institute, Cary, NC, USA). Significant differences between two and three groups were evaluated using Student’s t-test and the Steel–Dwass test, respectively. Correlation coefficients were calculated using the Spearman’s rho test. Statistical significance was set at *P* < 0.05.

## Supplementary Information

Supplementary Figures.

Supplementary Video 1.

Supplementary Video 2.

## Data Availability

Data are available from the corresponding author upon reasonable request.

## References

[1] Ruijs A, Jaquet J, Kalmijn S, Giele H, Hovius S (2005). Median and ulnar nerve injuries: A meta-analysis of predictors of motor and sensory recovery after modern microsurgical nerve repair. Plast. Reconstr. Surg..

[2] Scheib J, Höke A (2013). Advances in peripheral nerve regeneration. Nat. Rev. Neurol..

[3] Li L (2020). Remnant neuromuscular junctions in denervated muscles contribute to functional recovery in delayed peripheral nerve repair. Neural Regen. Res..

[4] Fu S, Gordon T (1997). The cellular and molecular basis of peripheral nerve regeneration. Mol. Neurobiol..

[5] Dun X (2019). Macrophage-derived Slit3 controls cell migration and axon pathfinding in the peripheral nerve bridge. Cell Rep..

[6] Cattin A (2015). Macrophage-induced blood vessels guide schwann cell-mediated regeneration of peripheral nerves. Cell.

[7] Matsui Y (2022). IL4 stimulated macrophages promote axon regeneration after peripheral nerve injury by secreting uPA to stimulate uPAR upregulated in injured axons. Cell. Mol. Life Sci..

[8] Chen P, Piao X, Bonaldo P (2015). Role of macrophages in Wallerian degeneration and axonal regeneration after peripheral nerve injury. Acta Neuropathol..

[9] Nadeau S (2011). Functional recovery after peripheral nerve injury is dependent on the pro-inflammatory cytokines IL-1β and TNF: Implications for neuropathic pain. J. Neurosci..

[10] Mokarram N, Merchant A, Mukhatyar V, Patel G, Bellamkonda R (2012). Effect of modulating macrophage phenotype on peripheral nerve repair. Biomaterials.

[11] Jiang X (2019). A review of low-intensity pulsed ultrasound for therapeutic applications. IEEE Trans. Biomed. Eng..

[12] Harrison A, Lin S, Pounder N, Mikuni-Takagaki Y (2016). Mode & mechanism of low intensity pulsed ultrasound (LIPUS) in fracture repair. Ultrasonics.

[13] Kubanek J, Shukla P, Das A, Baccus S, Goodman M (2018). Ultrasound elicits behavioral responses through mechanical effects on neurons and ion channels in a simple nervous system. J. Neurosci..

[14] Daeschler S (2018). Ultrasound and shock-wave stimulation to promote axonal regeneration following nerve surgery: A systematic review and meta-analysis of preclinical studies. Sci. Rep..

[15] Akhlaghi Z (2012). The effects of altered ultrasound parameters on the recovery of sciatic nerve injury. Iran Biomed. J..

[16] Jiang W (2016). Low-intensity pulsed ultrasound treatment improved the rate of autograft peripheral nerve regeneration in rat. Sci. Rep..

[17] Ito A (2020). Ultrasound therapy with optimal intensity facilitates peripheral nerve regeneration in rats through suppression of pro-inflammatory and nerve growth inhibitor gene expression. PLoS ONE.

[18] Kawai H, Ito A, Wang T, Xu S, Kuroki H (2022). Investigating the optimal initiation time of ultrasound therapy for peripheral nerve regeneration after axonotmesis in rats. Ultrasound Med. Biol..

[19] Zhou S (2008). Low intensity pulsed ultrasound accelerates macrophage phagocytosis by a pathway that requires actin polymerization, Rho, and Src/MAPKs activity. Cell Signal..

[20] da Silva Junior E (2017). Modulating effect of low intensity pulsed ultrasound on the phenotype of inflammatory cells. Biomed. Pharmacother..

[21] DeLeonibus A (2021). A meta-analysis of functional outcomes in rat sciatic nerve injury models. Microsurgery.

[22] Vannucci B (2019). What is normal? Neuromuscular junction reinnervation after nerve injury. Muscle Nerve.

[23] Hong Y (2022). Ultrasound stimulation improves inflammatory resolution, neuroprotection, and functional recovery after spinal cord injury. Sci. Rep..

[24] Feng S, Zhang P (2023). The significance of M1 macrophage should be highlighted in peripheral nerve regeneration. Histol. Histopathol..

[25] Shen Y, DeBellard M, Salzer J, Roder J, Filbin M (1998). Myelin-associated glycoprotein in myelin and expressed by Schwann cells inhibits axonal regeneration and branching. Mol. Cell. Neurosci..

[26] de Medinaceli L, Freed W, Wyatt R (1982). An index of the functional condition of rat sciatic nerve based on measurements made from walking tracks. Exp. Neurol..

[27] Lee J (2013). Functional evaluation in the rat sciatic nerve defect model: A comparison of the sciatic functional index, ankle angles, and isometric tetanic force. Plast. Reconstr. Surg..

[28] Weber R, Proctor W, Warner M, Verheyden C (1993). Autotomy and the sciatic functional index. Microsurgery.

[29] Wang T (2018). Functional evaluation outcomes correlate with histomorphometric changes in the rat sciatic nerve crush injury model: A comparison between sciatic functional index and kinematic analysis. PLoS ONE.

[30] Alant J (2013). The impact of motor axon misdirection and attrition on behavioral deficit following experimental nerve injuries. PLoS ONE.

[31] Lv D, Zhou L, Zheng X, Hu Y (2017). Sustained release of collagen VI potentiates sciatic nerve regeneration by modulating macrophage phenotype. Eur. J. Neurosci..

[32] McLean N, Verge V (2016). Dynamic impact of brief electrical nerve stimulation on the neural immune axis-polarization of macrophages toward a pro-repair phenotype in demyelinated peripheral nerve. Glia.

[33] Siemionow M (2011). Peripheral nerve defect repair with epineural tubes supported with bone marrow stromal cells: A preliminary report Ann. Plast. Surg..

[34] Yurie H (2017). The efficacy of a scaffold-free Bio 3D conduit developed from human fibroblasts on peripheral nerve regeneration in a rat sciatic nerve model. PLoS ONE.

