# Supplementary material for: Ultrasound therapy for a week promotes regeneration and reduces pro-inflammatory macrophages in a rat sciatic nerve autograft model
Source: Sci Rep. 2023 Jul 17;13:11494. doi: 10.1038/s41598-023-38630-8 (PMC10352352; doi:10.1038/s41598-023-38630-8)
Supplement: Supplementary file 1 — Supplementary Figures. [file 41598_2023_38630_MOESM1_ESM.pdf]

Ultrasound therapy for a week promotes regeneration and reduces pro-inflammatory macrophages in a rat sciatic nerve autograft model

Authors

Hideki Kawai[1,2], Akira Ito[1]*, Asuka Kawaguchi[1], Momoko Nagai-Tanima[1], Ryo Nakahara[1], Shixuan Xu[1], Hiroshi Kuroki[1]

Affiliations

[1] Department of Motor Function Analysis, Human Health Sciences, Graduate School of Medicine, Kyoto University, Kyoto, Japan

[2] Japan Society for the Promotion of Science, Tokyo, Japan

*Corresponding author

Akira Ito

Tel: + 81-75-751-3964

E-mail: ito.akira.4m@kyoto-u.ac.jp

Address: 53 Kawahara-cho, Shogoin, Sakyo-ku, Kyoto 606-8507, Japan

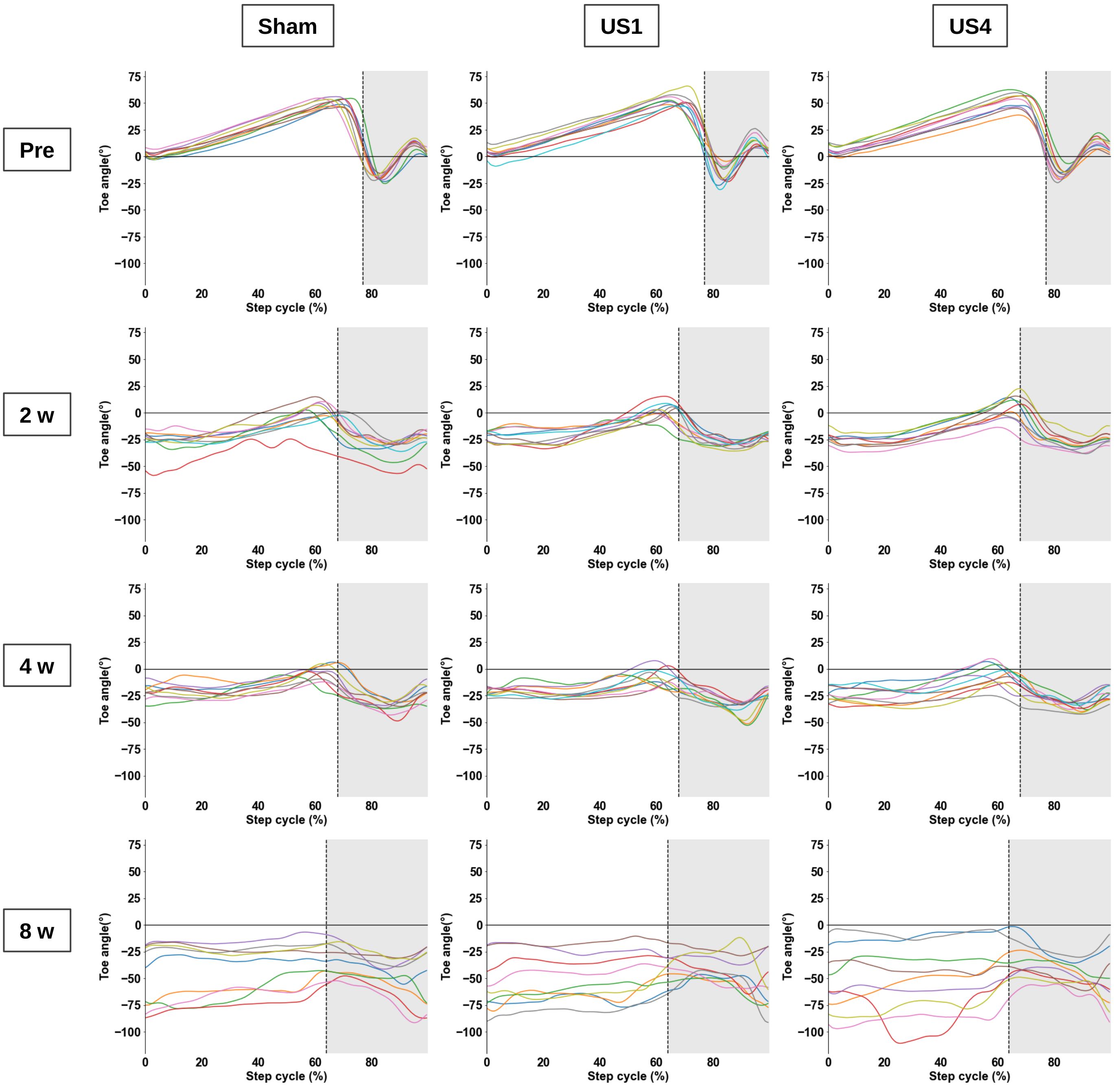

Supplementary Figure S1. Toe angles in all step cycles.
Each of the ten steps recorded using a three-dimensional motion capture apparatus was normalized from the toe contact (0%) to just before the subsequent toe contact (99.5%), and then the angles of the steps were averaged at each time point. Each line shows the data for a single rat (n = 9 for each group). Dashed lines indicate the timing of toe-off, and gray areas indicate the swing phase.

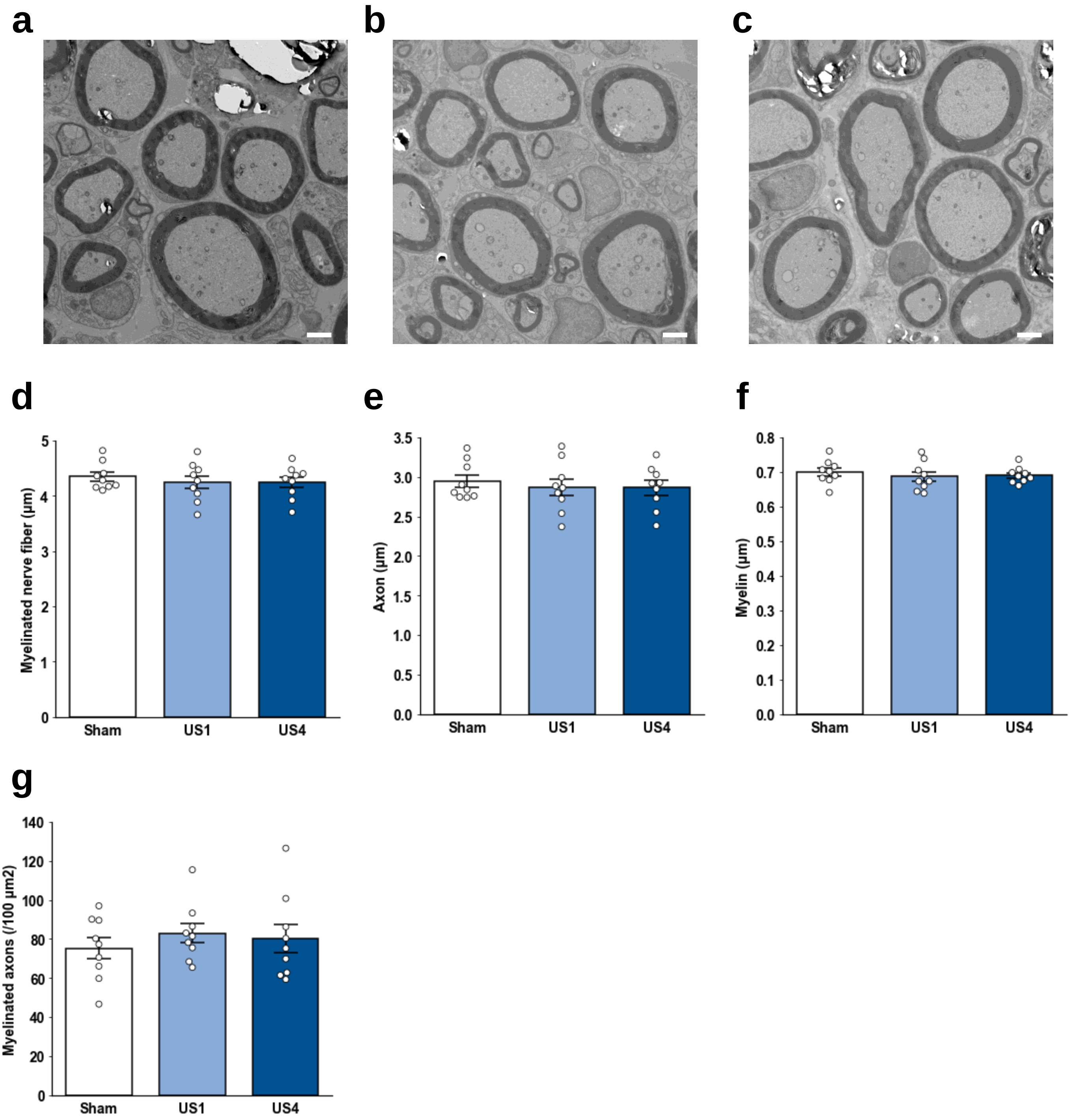

Supplementary Figure S2. The results of histomorphometry.
Representative images obtained by transmission electron microscopy of the sham (**a**), US1 (**b**), and US4 groups (**c**). (**d**) The evaluated diameter of myelinated nerve fibers and (**e**) axons, (**f**) myelin sheath thickness, and (g) the density of the myelinated axons was evaluated. Each plot shows individual values (n = 9 for each group). All scale bars = 2 μm.

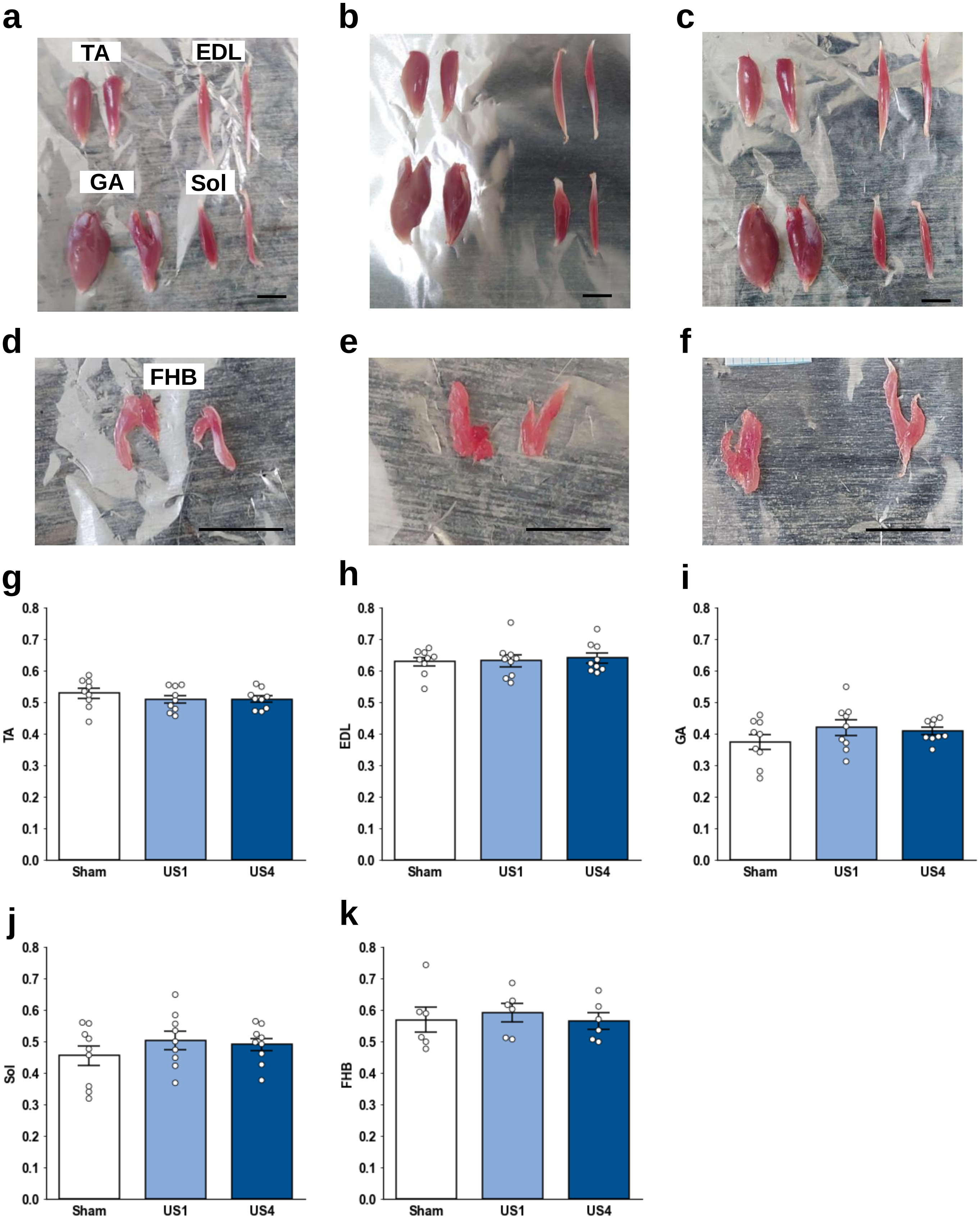

Supplementary Figure S3. The results of wet muscle weight analysis.
Representative images of the sham (**a**, **d**), US1 (**b**, **e**), and US4 groups (**c**, **f**). The muscles on the left and right sides indicate non-injured and injured sides, respectively. (**g**) The tibialis anterior (TA), (**h**) extensor digitorum longus (EDL), (**i**) gastrocnemius (GA), (**j**) soleus (Sol), and (**k**) flexor hallucis brevis (FHB) muscles are weighed and expressed as a ratio of the injured side to the non-injured side. Data are expressed as the mean ± standard error. Each plot shows individual values (n = 6 for FHB and n = 9 for the rest). All scale bars = 1 mm.

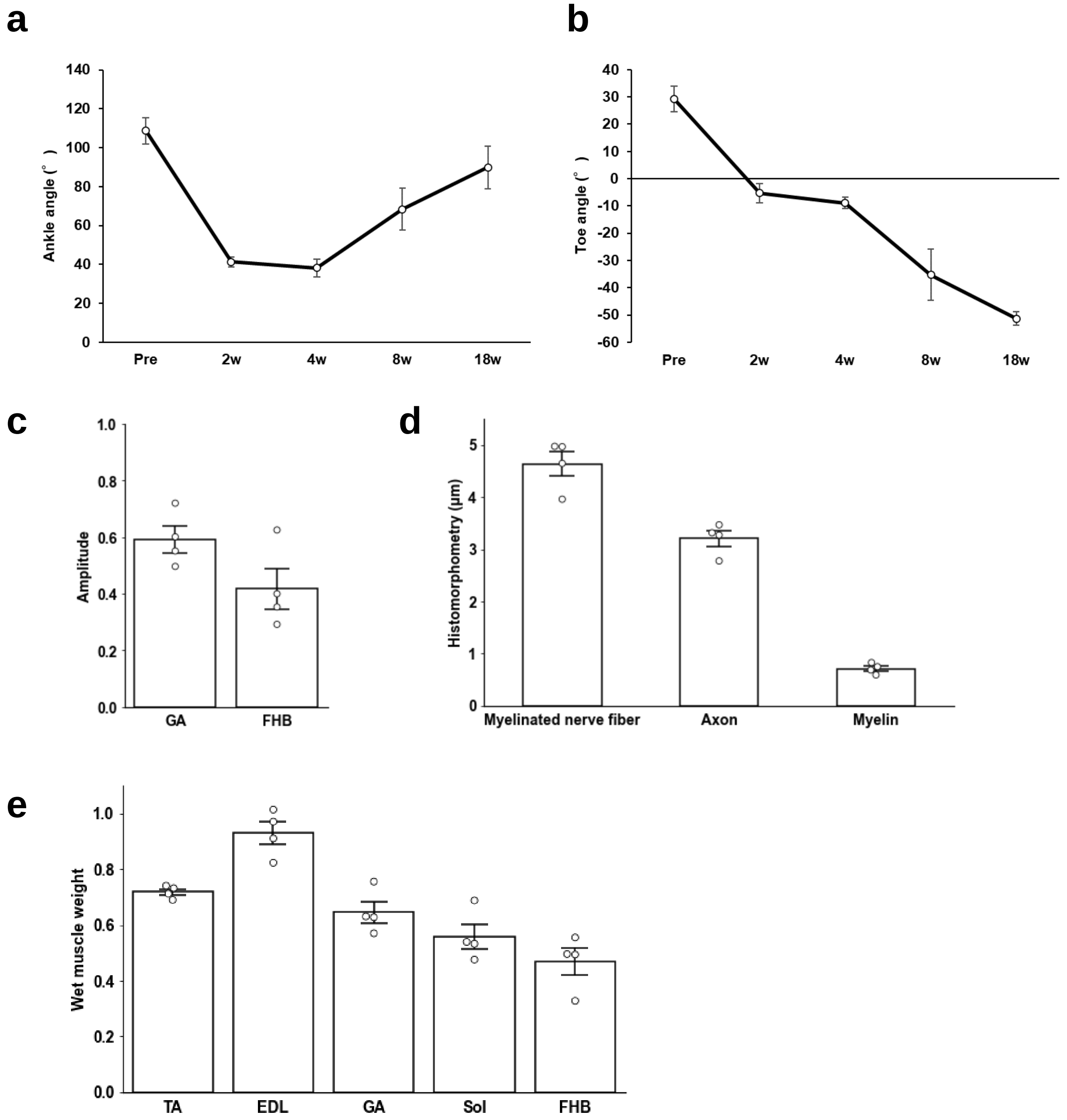

Supplementary Figure S4. No recovery in the gait pattern, even 18 weeks after autografting. (**a**) The ankle and (**b**) toe angles evaluated preoperatively and at 2, 4, 8, and 18 weeks after sciatic nerve autografting using another four rats. The ankle angles recovered, whereas the toe angles continued to decrease. (**c**) The amplitudes of compound muscle action potentials of the gastrocnemius (GA) and flexor hallucis brevis (FHB) at 18 weeks after autografting are expressed as the ratio of the injured side to the non-injured side. (**d**) Diameter of myelinated nerve fibers, axons, and myelin sheath thickness at 18 weeks after autografting. (**e**) The tibialis anterior (TA), extensor digitorum longus (EDL), GA, soleus (Sol), and FHB muscles weighed 18 weeks after injury and expressed as the ratio of the injured side to the non-injured side. Data are expressed as the mean ± standard error. Each plot shows individual values (n = 4).
